# Demographic history and genetic diversity of wild African harlequin quail (*Coturnix delegorguei delegorguei*) populations of Kenya

**DOI:** 10.1002/ece3.8458

**Published:** 2021-12-13

**Authors:** Stephen Ogada, Newton O. Otecko, Grace Moraa Kennedy, John Musina, Bernard Agwanda, Vincent Obanda, Jacqueline Lichoti, Min‐Sheng Peng, Sheila Ommeh

**Affiliations:** ^1^ Institute For Biotechnology Research Jomo Kenyatta University of Agriculture and Technology Nairobi Kenya; ^2^ State Key Laboratory of Genetic Resources and Evolution, Yunnan Laboratory of Molecular Biology of Domestic Animals Kunming Institute of Zoology Chinese Academy of Sciences Kunming China; ^3^ Sino‐Africa Joint Research Center Chinese Academy of Sciences Nairobi Kenya; ^4^ Department of Zoology National Museums of Kenya Nairobi Kenya; ^5^ Department of Veterinary Services Kenya Wildlife Service Nairobi Kenya; ^6^ Central Veterinary Laboratories Kabete State Department of Livestock Ministry of Agriculture, Livestock and Fisheries Nairobi Kenya

**Keywords:** admixture, demographic inference, genetic differentiation, genotyping‐by‐sequencing, mtDNA

## Abstract

Hunting wild African harlequin quails (*Coturnix delegorguei delegorguei*) using traditional methods in Western Kenya has been ongoing for generations, yet their genetic diversity and evolutionary history are largely unknown. In this study, the genetic variation and demographic history of wild African harlequin quails were assessed using a 347bp mitochondrial DNA (mtDNA) control region fragment and 119,339 single nucleotide polymorphisms (SNPs) from genotyping‐by‐sequencing (GBS) data. Genetic diversity analyses revealed that the genetic variation in wild African harlequin quails was predominantly among individuals than populations. Demographic analyses indicated a signal of rapid demographic expansion, and the estimated time since population expansion was found to be 150,000–350,000 years ago, corresponding to around the Pliocene–Pleistocene boundary. A gradual decline in their effective population size was also observed, which raised concerns about their conservation status. These results provide the first account of the genetic diversity of wild African harlequin quails of Siaya, thereby creating a helpful foundation in their biodiversity conservation.

## Graphical abstract

This study looks into the genetic diversity and demographic history of wild African harlequin quails using mtDNA and genotyping by sequencing data. The results of this work will provide a foundation useful in its protection and biodiversity conservation.

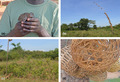

## 1 INTRODUCTION

The primary source of poultry protein for many rural households in Africa and other developing countries is chicken, though progressively, quails have become a vital supplement, increasing their socio‐economic value (MOLD, 2010). The African harlequin quail (*C*. *d*. *delegorguei*) is among the wild quail species commonly found in East and Southern Africa (Lewis & Pomeroy, 1989). Other wild quail species in the region include the common quail (*Coturnix coturnix)*, African blue quail (*Coturnix adansonii*), and rain quail (*Coturnix coromandelica*). However, the Japanese quail (*Coturnix coturnix japonica*) is the most common reared species in Kenya and other parts of the world, mainly kept for both meat and egg production (Nishibori et al., 2001). The ongoing illegal hunting of wild African harlequin quails by rural small‐scale farmers using traditional methods in Western Kenya is trending to unsustainable levels leading to declining numbers over time (Wamuyu et al., 2017). The seasonal migration of wild African harlequin quails into farmlands encourages their incessant capture for sale and consumption. At the moment, control measures to check on illegal and uncontrolled hunting are nonexistent, and little to no effort has been made to monitor and study their conservation status.

The domestication of Japanese quail during the late 19th century and early 20th century has contributed immensely toward improved poultry meat and egg production (Lukanov & Pavlova, 2020). Currently, the domestic Japanese quail can be found in many countries where they are selectively bred into meat and egg types (Jeke et al., 2018). The introduction of domestic Japanese quails in Africa and other developing countries has provided entrepreneurial opportunities and an alternative way of alleviating animal protein deficiency in their populations (Nasar et al., 2016). However, this introduced new challenges such as the uncontrolled introduction of immense exotic Japanese quail breeds in Kenya and other sub‐Saharan countries (Jeke et al., 2018). Considering that the domestic quail business bubble soon deflated, some of these exotic quail breeds were disposed of by frustrated farmers. Planned or accidental release of domestic quail individuals into the wild would bring adverse genetic changes to wild quail populations such as loss of genetic variation and altered population structures (Laikre et al., 2010). Admixture between the domestic and wild quail populations in some parts of the world has been observed where wild quails were found to be hybrids of the domestic Japanese quail (*C*. *Japonica*) and the wild common (*C*. *Coturnix*) quail (Amaral et al., 2007; Barilani et al., 2005; Chazara et al., 2010; Sanchez‐Donoso et al., 2012).

The wild African harlequin quail is endemic to Africa, but its population genetics and demographic history are largely unknown. Information on its genetic integrity is critical to its utilization and conservation. Illegal and unsustainable harvesting, coupled with the change in land use, habitat fragmentation, and the introduction of exotic breeds, are factors that might impact wild African harlequin quail genetics. Here, mitochondrial and genome‐wide variation patterns were used to assess wild African harlequin quail diversity and demographic history. In addition, tests were conducted to detect the presence of any wild African harlequin quail individuals admixed with the domestic Japanese quail.

## 2 MATERIALS AND METHODS

### 2.1 Sample collection and DNA extraction

**FIGURE 1:**
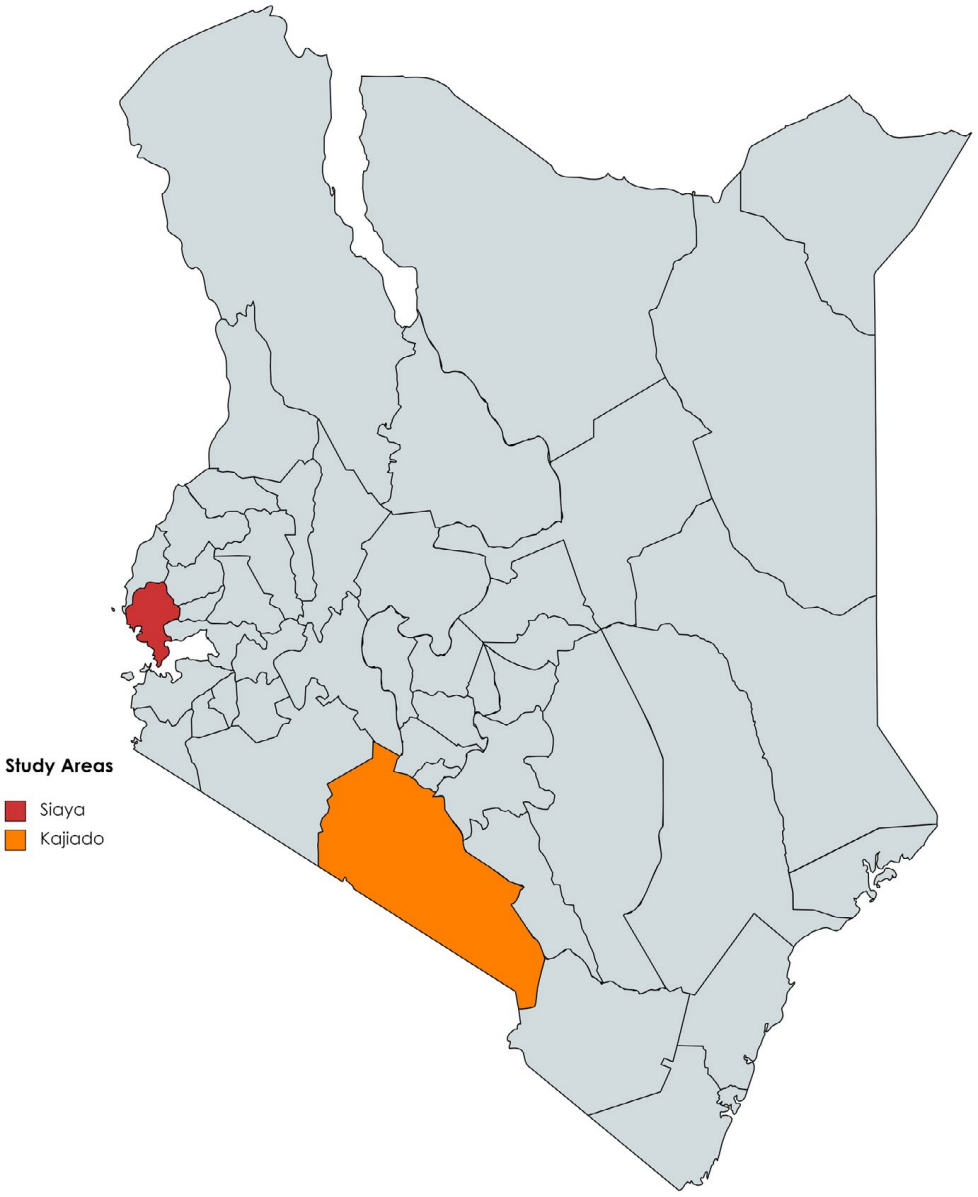
Sampling locations in Kenya

**FIGURE 2:**
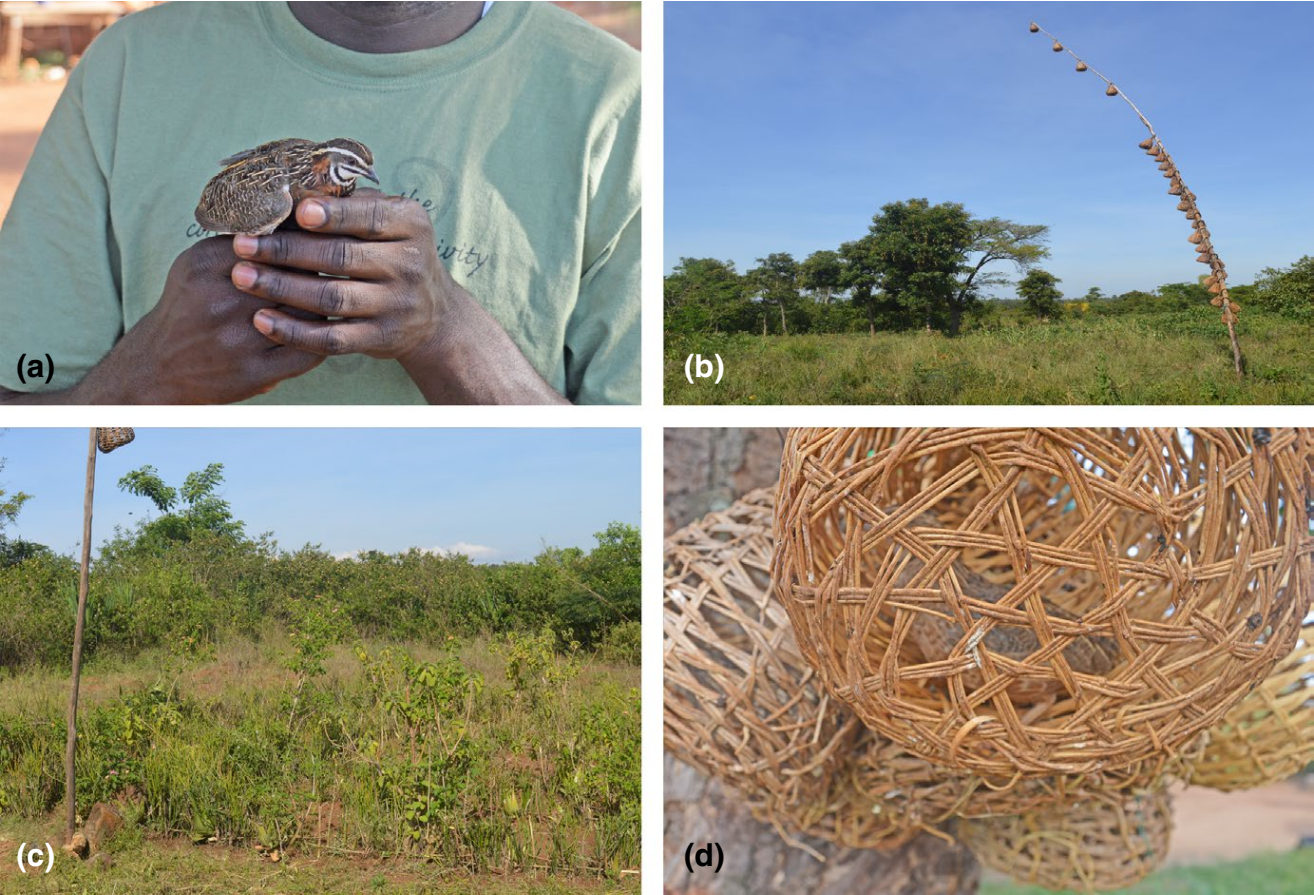
Wild African harlequin quail capture using traditional methods. (a) Wild African harlequin quail sample photograph. (b) Long poles containing bait quails. (c) Man‐made thicket with traps. (d) Captured wild African harlequin quails in woven baskets

One hundred quails were sampled for this study; 78 captured wild African harlequin quails (Siaya North = 24, Siaya Central = 28, Siaya South = 26) and 22 domestic Japanese quails from Siaya and Kajiado Counties of Kenya, respectively (Figure 1). This comprised an equal number of male and female individuals for every visited farm. Wild African harlequin quails were captured around homesteads using traditional methods described by Wamuyu et al. (2017). This involves using previously captured quails that are placed in woven baskets then tied to long poles as bait to lure other quails which are captured by traps set on the ground (Figure 2). Siaya County was selected due to the significant seasonal presence of wild African harlequin quails in the region, compared to others, and their interaction with rural small‐scale farmers. It lies in the Lake Victoria basin at an altitude of 1,134 meters above sea level, receiving an annual rainfall of 500–1000 mm with temperatures ranging 17.1–29.4°C (Moraa et al., 2015). In contrast, Kajiado County is a peri‐urban area where purebred domestic Japanese quails are more commonly reared under intensive (caged) production system. Whole blood was drawn from the wing vein into 2‐ml cryotubes containing 70% alcohol and preserved at −80°C. Genomic DNA was extracted from the whole blood samples using the phenol–chloroform method (Sambrook & Russell, 2006).

### 2.2 Mitochondrial DNA amplification and sequencing

A 760bp mitochondrial DNA control region fragment was amplified via polymerase chain reaction (PCR) using primers AV1F2 and CR1b (Mwacharo et al., 2011) in 56 wild African harlequin and 17 Japanese quails. The amplicons were then sequenced using Applied Biosystems 3730xl DNA Analyzer (Life Technologies) following the manufacturer's protocol. Forward and reverse sequences of each sample were edited using SeqMan Pro version 7.1.0 (DNASTAR, Inc.) and used to generate a consensus sequence. Sequence alignment was done using Clustal X version 2.1 (Thompson et al., 1997) and MUSCLE version 3.6 (Edgar, 2004). Highly conserved regions of the sequences were trimmed, and a variable 347bp fragment was retained for further analysis.

### 2.3 GBS library preparation and sequencing

A GBS predesign experiment approach was used to evaluate the enzymes and sizes of restriction fragments using training data. The following criteria were applied:

1. the number of tags must be suitable for the specific needs of the research project;
2. the enzymatic tags must be evenly distributed through the sequences to be examined;
3. repeated tags should be avoided.

These considerations improved the effectiveness of GBS for the dataset at hand. A strict length range was selected to maintain the sequence depth uniformity of different fragments (~50 bp).

We then constructed the GBS library in accordance using the predesigned scheme. Genomic DNA was incubated at 37℃ with *Mse*I (New England Biolabs, NEB), T4 DNA ligase (NEB), ATP (NEB), and *Mse*I Y adapter N‐containing barcode. The restriction–ligation reactions were heat‐inactivated at 65℃ and then digested with the *Hae*III (GGCC) restriction enzyme at 37°C. The restriction ligation samples were purified with Agencourt AMPure XP (Beckman, United States). Polymerase chain reaction (PCR) was conducted using the purified samples, the Phusion Master Mix (NEB, United States) universal and index primers, and i5 and i7 sequences. The PCR products were purified using Agencourt AMPure XP, pooled, and electrophoresed on a 2% agarose gel. A Gel Extraction Kit (Qiagen, Germany) was used to isolate 375–400 basepair fragments (with indexes and adaptors). These fragments were purified using Agencourt AMPure XP, and the resulting products were diluted for sequencing. Then, paired‐end sequencing of the selected tags was performed using the Illumina NovaSeq high‐throughput sequencing platform at the Novogene Bioinformatics Technology Company, China.

### 2.4 Sequence data processing and SNP calling

Raw reads were processed through a series of quality control procedures which involved removing reads with ≥10% unidentified nucleotides (*N*), with >50% bases with a Phred quality <5, with >10 nucleotides alignment to the adapter, with >10% mismatches, and that contain the *Hae*III enzyme sequence.

Burrows‐Wheeler Aligner (BWA) version 0.7.17 was used to align the retained reads against the *Coturnix Japonica* 2.0 genome (Assembly accession number GCF_001577835.1) with the parameters “mem ‐t 4 ‐k 32 ‐M” (Li & Durbin, 2009). Variant calling was performed using SAMtools version 1.11 *mpileup* command (Li et al., 2009) in conjunction with BCFtools version 1.11 *call* command (Li, 2011). Variant filtering was performed by restricting the dataset to biallelic SNPs found in at least 80% of samples, with a minimum depth of 2 reads, minimum Phred score of 30, and minimum minor allele frequency (MAF) of 0.05 using VCFtools version 0.1.13 (Danecek et al., 2011).

### 2.5 Genetic diversity and population structure

Mitochondrial genome haplotype diversity was assessed using DnaSP version 5.10.1 (Librado & Rozas, 2009). The phylogenetic relationship between the study mtDNA haplotypes and GenBank reference mtDNA haplotypes (*C*. *japonica* and *C*. *coturnix*) was estimated using a median‐joining network and a maximum likelihood tree generated with network version 5.0 (Bandelt et al., 1999) and MEGA version 10.2 (Kumar et al., 2018), respectively.

Molecular variation among populations test (*F_ST_*) and principal component analysis (PCA) of the genotyping‐by‐sequencing SNP dataset were used to assess the distribution of genetic variation among wild African harlequin quail individuals and populations. Both tests were performed using SNPRelate R package version 1.22.0 (Zheng et al., 2012) with additional filtering based on linkage disequilibrium (*r* ^2^ = 0.2). To investigate the genetic ancestry of the wild African harlequin quail individuals and admixture between the wild and domestic quail species, a maximum‐likelihood‐based clustering algorithm in ADMIXTURE version 1.3.0 was applied (Alexander & Lange, 2011) for *K* values 1 to 10. *K*‐value with the lowest cross‐validation (CV) error value was considered most optimal.

### 2.6 Demographic analysis

MtDNA neutrality tests (Tajima's *D* and Fu's *F*s) and mismatch analysis (sum of squares deviation (SSD), raggedness index (rag.), theta0 (*θ*_0_), theta1 (*θ*_1_), and Tau (*τ*)) were calculated using Arlequin version 3.5.2.2 (Excoffier & Lischer, 2010) to check for signals of demographic expansion through investigating mismatch distributions of pairwise differences among mtDNA haplotypes. Time since population expansion (*t*) was calculated using the equation *τ* = 2 *μLt*, where *τ* is the tau value, *μ* is the substitution rate, and *L* is the length of the sequence (Aoki et al., 2018). Like most other avian species, the mitochondrial genome mutation rate of wild African harlequin quail or other quail species, especially for the control region, is unknown. Therefore, the commonly used mitochondrial genome mutation rate of 2% per million years (Myr) was applied in this study (Nabholz et al., 2016). The divergence times between African harlequin quails and related quail species such as the Japanese and common quails were estimated using the TimeTree database (Kumar et al., 2017).

To examine the recent demographic history of the wild African harlequin quail using the SNP dataset, all populations were combined, and easySFS (Gutenkunst et al., 2009) was used to generate folded site frequency spectrum (SFS) information. The historical population size changes were then estimated using Stairway plot version 2.1 (Liu & Fu, 2015). The stairway plot has proved applicable to low‐depth sequencing and reduced‐representation sequence data (Liu & Fu, 2020). The mutation rate of the wild African harlequin quail is unknown, but since the mean mutation rate of most avian genomes ranges between 1.23 and 2.21 × 10^−9^ site^−1^ year^−1^, an intermediate rate observed in chicken (1.91e‐9) and a generation time of 1 year was assumed (Nam et al., 2010).

## 3 RESULTS

### 3.1 SNP identification and MtDNA haplotype analyses

More than 95% of the wild African harlequin quail sequenced reads in all individuals were mapped to the Japanese quail reference genome, thereby ascertaining confidence in the SNP identification process. A total of 8,237,001 variants were called in 96 individuals, and after variant filtering, 119,339 SNPs were retained for further analysis.

The 56 mtDNA control region sequences of the wild African harlequin quails revealed 32 haplotypes in total (haplotype 1–32), which translated to a haplotype diversity of 0.949±0.016, whereas only one haplotype (haplotype 33 farm‐reared) was observed among the 17 domestic Japanese quails mtDNA control region sequences. These generated haplotypes were then used in subsequent analysis.

### 3.2 Genetic diversity and population structure analysis

**FIGURE 3:**
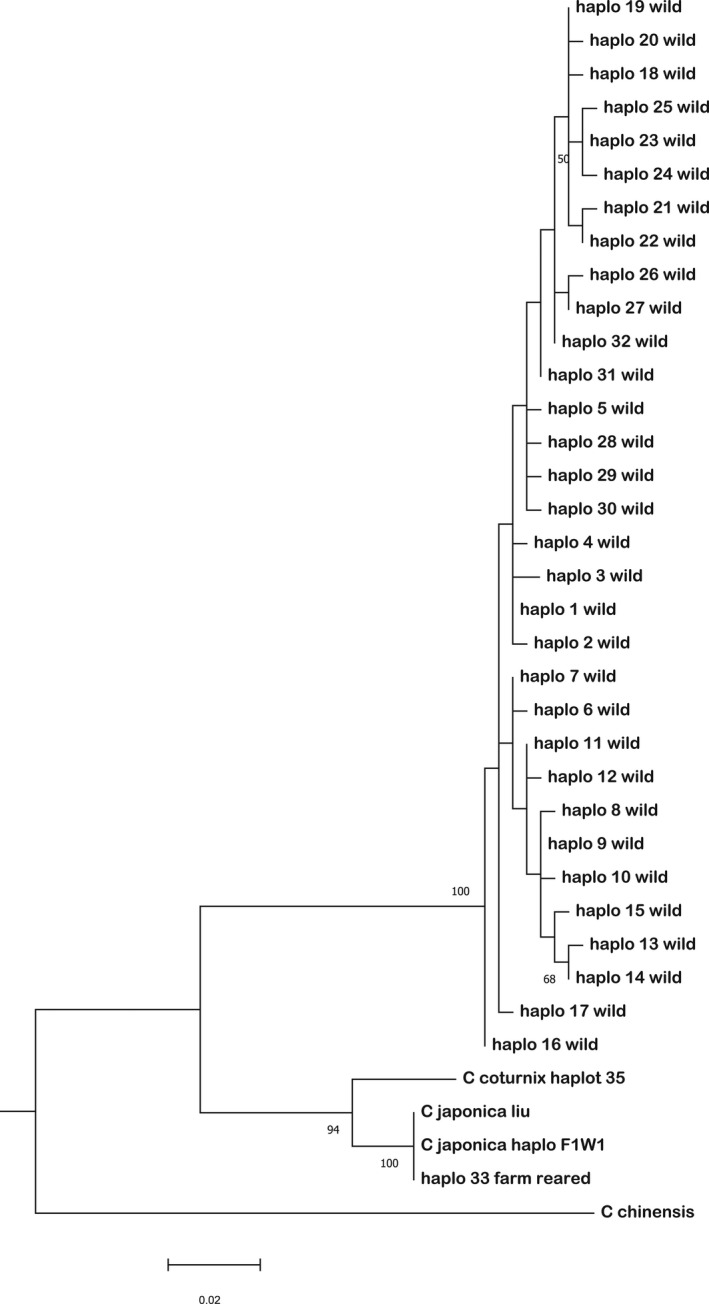
A maximum‐likelihood tree showing the phylogenetic relationships between wild African harlequin quails (haplo 1 wild – haplo 32 wild) and other *Coturnix* species

**FIGURE 4:**
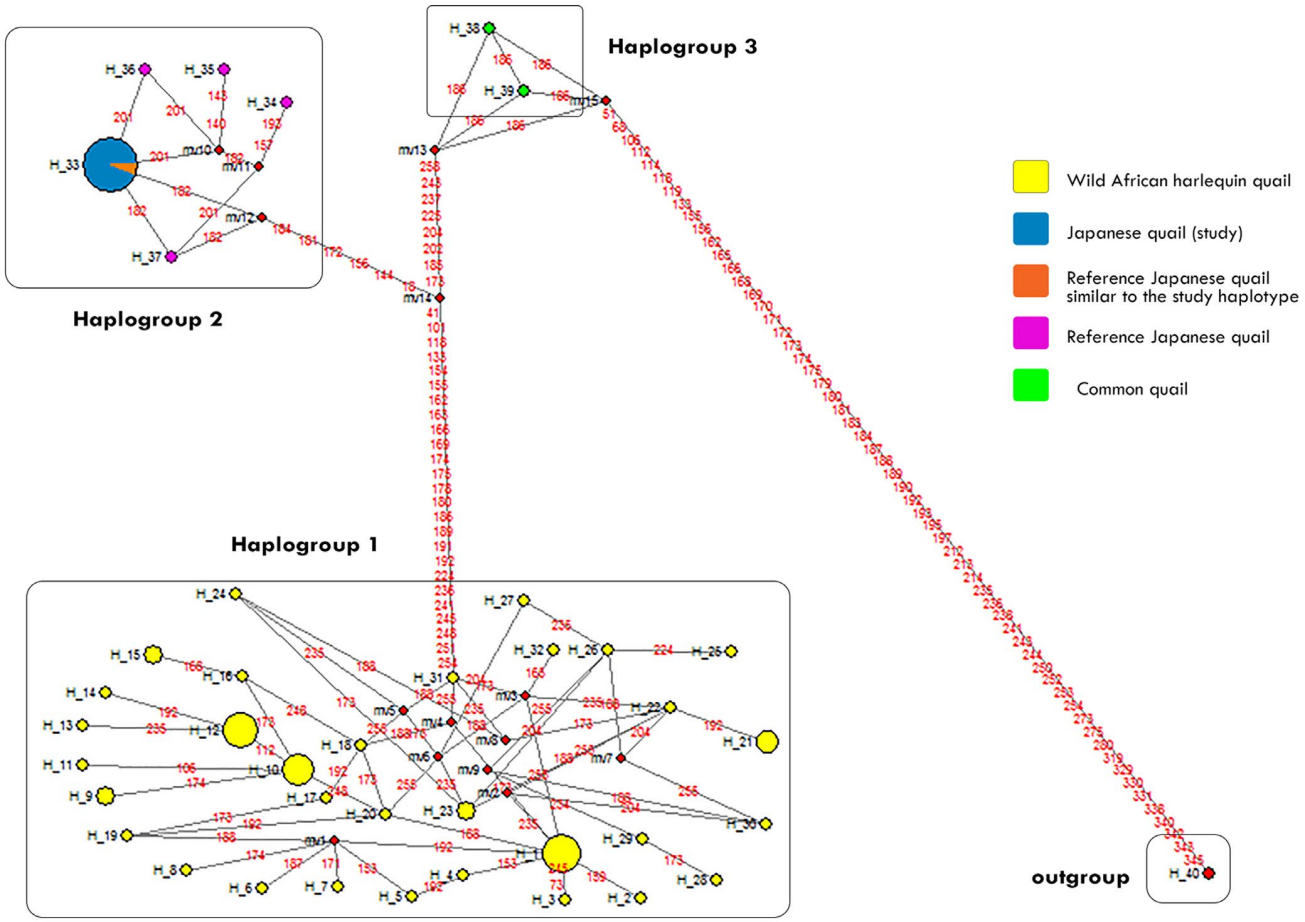
Median‐joining phylogenetic network constructed for quail mtDNA haplotypes. Circled areas show the haplotype frequencies

Out of the 32 wild African harlequin quail mtDNA haplotypes observed, haplotype 1 was the most common and present in 9 individuals. In contrast, the majority of the haplotypes were only present in one individual each (singletons). None of the other reference quail mtDNA haplotypes (Table S1) clustered together with the wild African harlequin mtDNA haplotypes; therefore, no haplotype sharing was observed (Figure 3). The median‐joining network of our study haplotypes together with reference quail mtDNA haplotypes (*C*. *japonica* and *C*. *coturnix*) from GenBank formed three haplogroups (Figure 4). All the wild African harlequin quail mtDNA haplotypes (yellow) clustered under haplogroup 1, whereas haplogroup 2 contained the single domestic Japanese quail mtDNA haplotype from this study (blue), reference Japanese quail mtDNA haplotype that was similar to the Japanese quail mtDNA haplotype of this study (orange) and other reference Japanese quail mtDNA haplotypes (purple) from GenBank (Table S2). Haplogroup 3 contrarywise contained reference common quail mtDNA haplotypes (green). The king quail (*C*. *chinensis*) mtDNA haplotype was the chosen outgroup.

**FIGURE 5:**
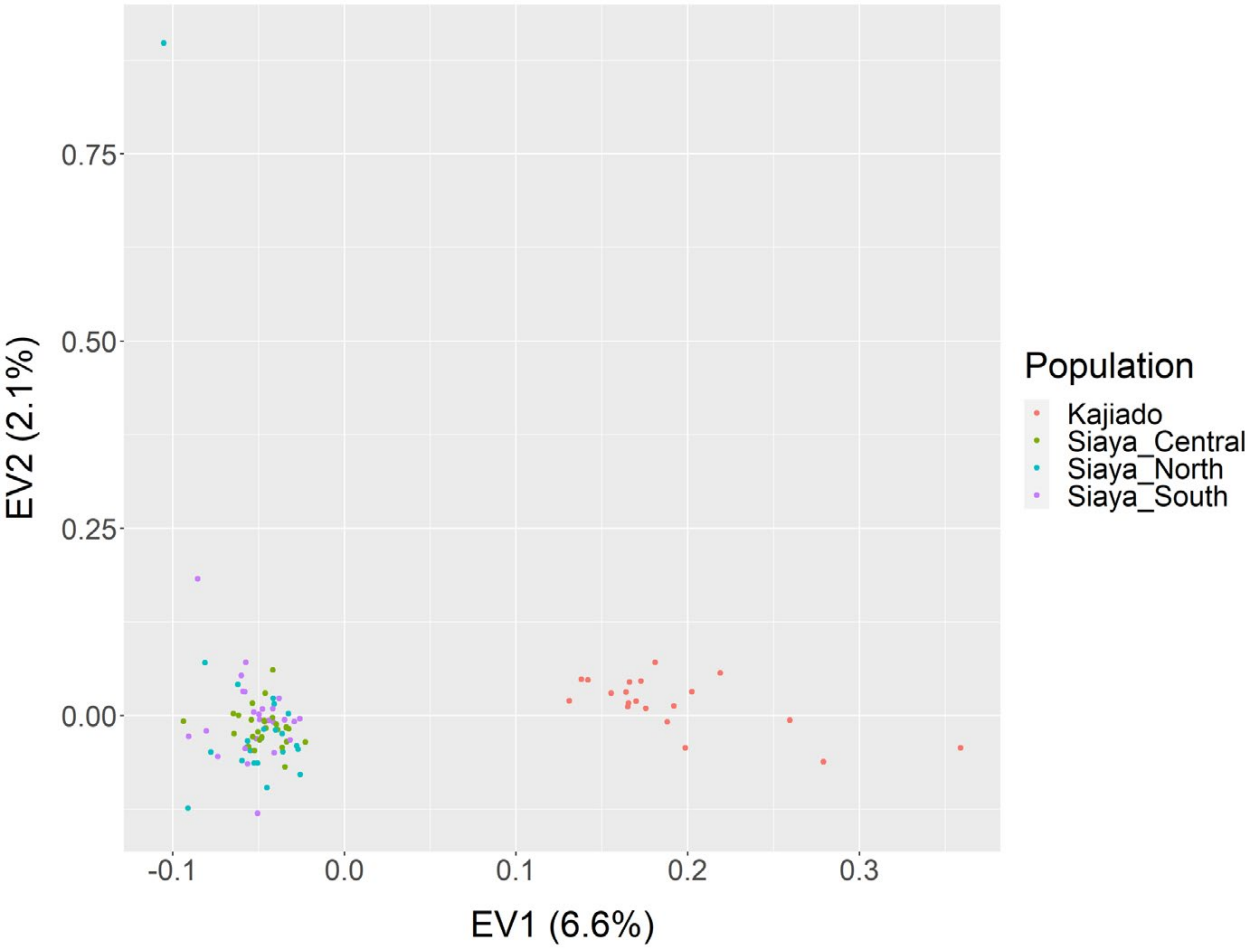
Principal component analysis of wild African harlequin quail populations (Siaya_North, Siaya_Central, and Siaya_South) and domestic Japanese quail (Kajiado)

Principal component analysis showed no signs of population structuring in wild African harlequin quails of Siaya County as most individuals were grouped in a single cluster (Figure 5). The first and second eigenvectors (EV) accounted for 6.6% and 2.1% of the total observed variation, respectively. This observation was also supported by a low average pairwise *F*_ST_ value of 0.000075, signifying slight genetic variation among the three sampled populations. Overall, the wild African harlequin and domestic Japanese quails were separated into two distinct clusters.

**FIGURE 6:**
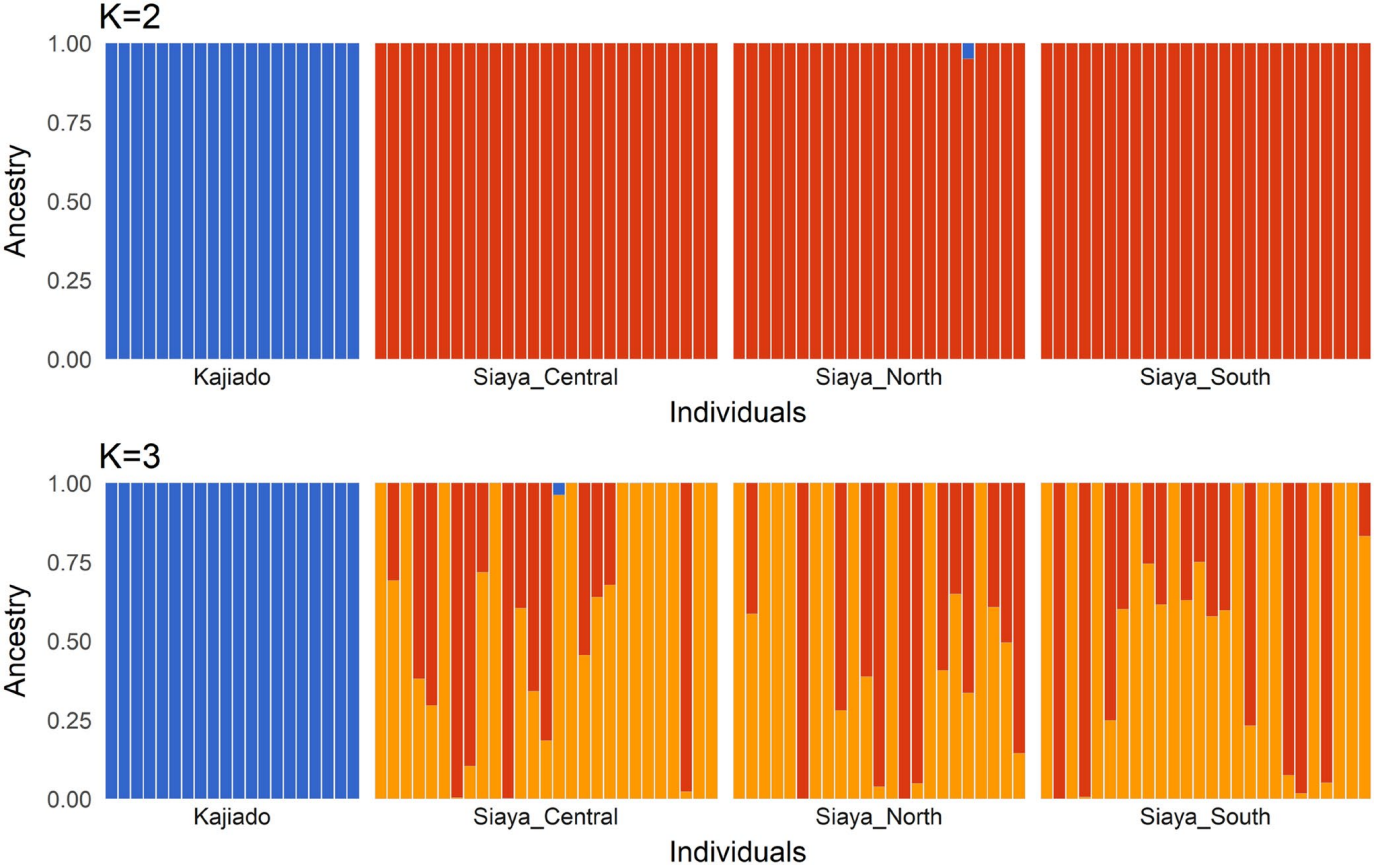
Admixture plot showing the relationship between wild African harlequin (Siaya) and domestic Japanese quails (Kajiado) at ancestry number (*K*) = 2–3

Admixture results were similar to PCA as the wild African harlequin quails were separated from domestic Japanese quails at *K* = 2, which had the least cross‐validation error (Figure S1). However, one wild African harlequin quail sample exhibited low levels of shared genetic ancestry with Japanese quail at *K* = 2, an observation that was not supported by other *K* values (Figure 6).

### 3.3 Demographic history

The estimated mean Tajima's *D* (−0.424 ± 0.366, *p* > .05) and Fu's *F*s (−7.67 ± 2.56, *p* < .05) values based on the mtDNA dataset were both negative for the wild African harlequin quail populations of Siaya County, indicating a signal of demographic expansion and/or positive selection. This observation was further supported by the unimodal mismatch distribution (Figure S2) and the small but nonsignificant SSD and Raggedness index that was 0.00573 ± 0.00170 (*p* > .05) and 0.0343 ± 0.00571 (*p* > .05), respectively. The population expansion size values (*θ*_0_ and *θ*_1_) were 0.00234 and 1169.888, respectively, also indicating a high population expansion. The tau (*τ*) value for the estimated age of expansion was 3.58 ± 0.504 (4.88–2.12) at a 95% confidence interval (CI). This translated to an estimated time since population expansion of 257,000 ka (Thousand years ago), 95% CI 150–350 ka, corresponding to around the Pliocene–Pleistocene boundary. The molecular divergence time between the African harlequin, Japanese, and common quails was estimated by the TimeTree database to range between 5.13 and 17.60 Ma (million years ago). However, the estimated median time was 11.03 Ma during the Miocene epoch of the Neogene period.

**FIGURE 7:**
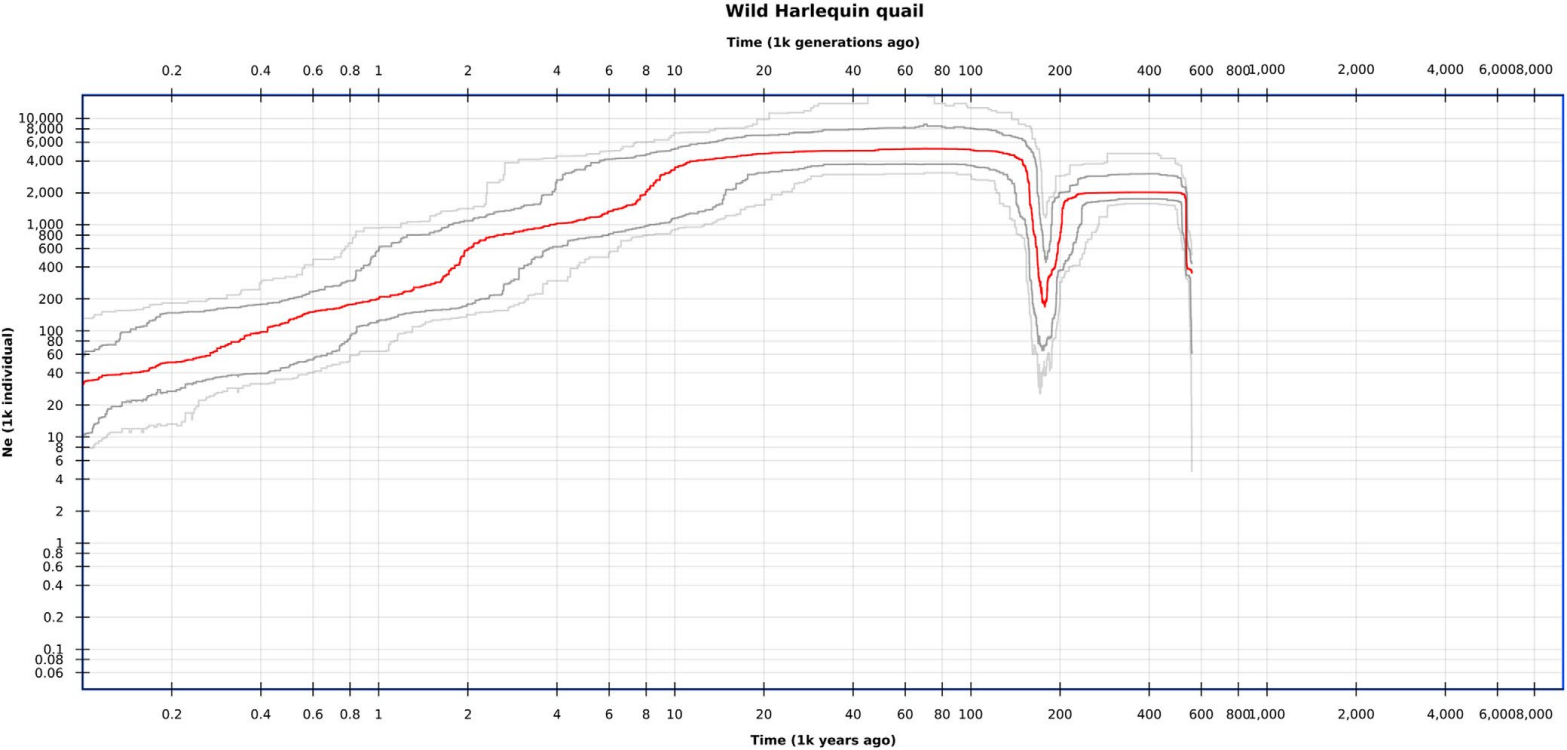
Stairway plot showing historical changes in effective population size of Siaya wild African harlequin quails

The demographic history and effective population size changes of the wild African harlequin quail of Siaya County were displayed on a stairway plot (Figure 7). The folded site frequency spectrum information based on genotyping by sequencing SNP data revealed a population bottleneck that occurred approximately 180 ka followed by population expansion then a gradual decline in the effective population size to the present.

## 4 DISCUSSION

Several genetic diversity studies have been conducted on domestic Japanese and wild common quails using mtDNA and microsatellite markers (Amaral et al., 2007; Barilani et al., 2005; Chazara et al., 2010; Puigcerver et al., 2014; Sanchez‐Donoso et al., 2012). However, genetic information on the wild African harlequin quail is lacking. In this study, we managed to assess the genetic diversity and demographic history of wild African harlequin quail populations of Siaya County using mtDNA d‐loop data and SNP information generated through genotyping by sequencing approach. There has been an uncontrolled introduction of domestic Japanese quail breeds into Kenya and a noticeable population size reduction of wild African harlequin quail numbers in parts of Western Kenya over the years. There have also been suspected cases of domestic Japanese quail release into the wild but so far, no credible evidence has been provided yet. Before any reasonable management practices are adopted to allow for proper utilization of the wild African harlequin quail as a resource and increase the effectiveness of conservation activities, more information with respect to the genetic diversity, demographic history, and population structure is required.

### 4.1 Genetic diversity and population structure

The seasonal migration of wild birds such as the wild African harlequin quails affects their population size and structure (Newton, 2006). Since there is little information about the seasonal migration patterns of wild African harlequin quails, sampling individuals from a similar deme or multiple demes of a single population is highly likely. This could explain why the genetic variation of wild African harlequin quails was predominantly among individuals and less among populations. The wild African harlequin quails were, however, separated from domestic Japanese quails into two distinct clusters. This difference is expected, as the two species have an estimated divergence time between 5.13 and 17.60 Ma (Jetz et al., 2012; Seabrook‐Davison et al., 2009; Stein et al., 2015).

The mtDNA haplotype and phylogenetic relationship analyses were also able to show the genetic variation among the wild African harlequin quail individuals, their similar ancestry, and their distinction from other quail species. In contrast, only a single mtDNA haplotype was detected among sampled Japanese quail individuals, which was consistent with low genetic diversity within this domesticated population. MtDNA haplotype sharing between wild and domestic quails has previously been used to detect hybridization (Barilani et al., 2005). However, there was no mtDNA haplotype sharing between wild African harlequin quails of Siaya County and Japanese quail individuals, separating the two species into different haplogroups. Even though no mtDNA haplotype sharing was observed, the wild African harlequin quail individual that showed evidence of low levels of Japanese quail ancestry in ADMIXTURE analysis conducted on SNP data still lacks strong evidence to suggest hybridization in the wild. The observed apparent admixed nature of the individual was not noted in other values of *K* and could be an artifact of the analysis. In addition, there are no known hybrids of *C*. *d*. *delegorguei* and *C*. *c*. *japonica* that exist in the wild hence further investigation involving a larger sample size is required.

These findings further alleviate concerns of possible extreme hybridization and introgression present in the local wild African harlequin quail populations of Siaya County since the excessive introduction of domestic Japanese quail breeds into the country. Detecting hybridization is crucial as it has been linked to reduced or absent migratory behavior in wild quail populations (Barilani et al., 2005). Currently, there is no reason to suspect the artificial release of domestic Japanese quail breeds into the wild as was the concern.

### 4.2 Demographic history

The demographic history of the wild African harlequin quails of Siaya was outlined on a stairway plot from 560,000 years ago. The population bottleneck event that was observed ca. 180 ka is consistent with climate change during the Pliocene–Pleistocene boundary where climate oscillations resulted in range contraction of species during dry arid climate periods (deMenocal, 1995; Hewitt, 2004). The observed bottleneck is also consistent with the emergence of *Homo sapiens* in East Africa some 200,000 years ago (McDougall et al., 2005; White et al., 2003) which was associated with updated and improved technology (Bradfield et al., 2020; Hallett et al., 2021). African hominins were known for hunting terrestrial prey and the development of advanced tools facilitated the incorporation of animal protein into their diets (Faith, 2014). However, the time period when early hominins became skilled hunters is still debatable (Ferraro et al., 2013; Marean & Assefa, 1999).

Both mtDNA analysis and the stairway plot suggested that wild African harlequin quail populations of Siaya experienced a population expansion soon after the bottleneck. The negative mtDNA Tajima's *D* and Fu's *F*s test values, and the demographic expansion estimates all signaled a large‐scale demographic expansion of the wild African harlequin quail populations of Siaya County. The unimodal mismatch distribution that was also detected when the observed nucleotide‐site differences between pairs of mtDNA haplotypes were computed is characteristic of a population that has experienced demographic expansion (Rogers & Henry, 1992). This could be through a range expansion with high levels of migration between neighboring demes that is commonly observed in wild quails that migrate seasonally (Excoffier, 2004; Ray et al., 2003). In addition, the demographic expansion parameter τ and stairway plot analysis were both used to infer time since population expansion and that estimate was placed around the quaternary glaciation period. Glacial changes during this period promoted the general cooling of African climates, and East African vegetation shifted from closed canopy to open savannah and grassland vegetation starting in the mid‐Pliocene (deMenocal, 1995, 2004). Grasslands, bushy areas, and croplands serve as habitats for many wild quail species, including the African harlequin quail, and this may explain their expansion during that period. In East Africa, vegetation changes and the development of periodical cooler and drier climatic conditions after 2.8 Ma greatly influenced species distribution and genetic isolation that further intensified after 1.7 and 1.0 Ma (deMenocal, 1995). Oscillations between wetter and drier climates have been found to repeatedly promote cycles of range contraction and expansion (Lorenzen et al., 2010). This may also explain the seasonal presence of wild African harlequin quails following the long and short rainy seasons (Wamuyu et al., 2017). Their sudden availability and increased numbers are also encouraged by food availability, especially after the rainy season, where grain farms create suitable habitats.

The effective population size of wild African harlequin quails of Siaya has been declining over the last 20,000 years, which coincides with climate change in East Africa, the massive late Pleistocene changes in range and abundance of smaller vertebrates (Steele, 2007) and the extinction of megafauna (50 ka −10 ka) (Van Der Kaars et al., 2017; Stuart, 1999). Climate change during the last glacial maximum (~20 ka) has been linked to a decline in the effective population size of several species in Southern and tropical Africa (Sithaldeen et al., 2015). Changes in temperature and precipitation were found to have a profound effect on food supply, reproduction, and the migration patterns of birds (Carey, 2009). In addition, this decline began after hydroclimatic changes that favored *Homo sapiens* demographic expansion and dispersal to other parts of the world approximately 70–60 ka (Rito et al., 2019; Schaebitz et al., 2021). It is hypothesized that during this period, human hunters were responsible for the massive extinction events and habitat destruction as they spread from Africa to other continents (Faith, 2014; Martin, 1967). However, the role played by humans in the late Pleistocene extinction events is still debated (Nagaoka et al., 2018).

Over the past 200,000 years, human occupation in East Africa has contributed to regional vegetation and habitat change (Schaebitz et al., 2021). This change was further facilitated by the transition from hunting and gathering to agriculture where rainforests and woody vegetation in the savanna were slowly converted into farms and pasturelands (Patin et al., 2014). Wild quails survive away from protected areas and end up in farms and pasturelands, where they are commonly hunted, thus also contributing to their population changes over time (Gatesire et al., 2014). Traditional hunting of wild African harlequin quails involves using captured females to attract males (Wamuyu et al., 2017). This leads to the capture of more males than females, thereby affecting the breeding sex ratio, which impacts the effective population size. This could also explain the disappearance of wild African harlequin quails from certain neighboring regions and their reduction in number within Siaya County, where they were usually present in very high numbers compared to other regions.

Wild African harlequin quails were initially present in most parts of Western Kenya. However, due to climatic change, intensive human activities such as overhunting and destruction of their habitats, parts of Siaya, Kisumu, and Homa Bay counties have become potential refugial areas. These activities influence and have contributed to migratory behavior changes and population decline of not only wild African harlequin quails but also other migratory bird species (Hewson et al., 2016). The wild African harlequin quails of Siaya County seemed to be concentrated in regions near the lake due to the presence of untouched natural vegetation unlike the mainland. So far, no comprehensive phylogeographic and climatic oscillation studies have been done on wild African harlequin quails or other wild bird species in the Lake Victoria basin. Therefore, the geographical impact of Lake Victoria on species diversity is still understudied. Even though this study concentrated on the wild African harlequin quail populations of Siaya, additional sampling sites could reveal if there is any range‐wide population structure and how interconnected different populations are.

## 5 CONCLUSION

The use of mitochondrial DNA and genotyping by sequencing methods provided an overview of the genetic diversity of wild African harlequin quail populations of Siaya County. We did not detect any population structure between individuals in our study regions; however, more study sites are recommended to fully capture individuals that follow different migratory routes. We found no evidence of hybridization between wild African harlequin and domestic Japanese quails in the Siaya region. A noticeable decline in the number of wild African harlequin quails in the region has been a source of concern in line with their conservation status. This study has provided the necessary information required for decision‐making regarding their preservation and utilization as a source of protein. Even though the harlequin quail subspecies (*C*. *d*. *delegorguei*, *C*. *d*. *histrionica*, and *C*. *d*. *arabica*) conservation status are listed collectively under the “least concern” category according to the International Union for Conservation of Nature (IUCN), more studies aimed at monitoring their distribution and effective population size changes need to be conducted, especially in areas where the harlequin quail is considered as a complementary poultry protein source.

## CONFLICT OF INTEREST

The authors declare no conflict of interest.

## AUTHOR CONTRIBUTIONS

**Stephen Ogada:** Formal analysis (lead); Methodology (equal); Validation (equal); Writing – original draft (lead); Writing – review & editing (equal). **Newton O. Otecko:** Methodology (equal); Validation (equal); Writing – review & editing (equal). **Grace Moraa Kennedy:** Formal analysis (supporting); Validation (equal); Writing – review & editing (equal). **John Musina:** Validation (equal); Writing – review & editing (equal). **Bernard Agwanda:** Validation (equal); Writing – review & editing (equal). **Vincent Obanda:** Validation (equal); Writing – review & editing (equal). **Jacqueline Lichoti:** Supervision (supporting); Validation (equal); Writing – review & editing (equal). **Min‐Sheng Peng:** Formal analysis (supporting); Validation (equal); Writing – review & editing (equal). **Sheila Ommeh:** Conceptualization (lead); Funding acquisition (lead); Investigation (lead); Methodology (equal); Project administration (lead); Resources (lead); Supervision (lead); Validation (equal); Writing – original draft (supporting); Writing – review & editing (equal).

## ETHICAL APPROVAL

This study received ethical clearance from the Kenya Wildlife Service under permit number KWS/BRM/5001 to sample wild African harlequin quails and a “no objection for the research” from the Director of Veterinary Services, Ministry of Agriculture, Livestock and Fisheries in Kenya under permit number RES/POL/VOL.XXVII/162 to sample domestic quails.

## Supporting information

Figure S1

Figure S2

Tables S1 and S2

## Data Availability

Wild African harlequin quail mitochondrial DNA D‐loop haplotype sequences were archived in the National Centre for Biotechnology Information (NCBI) repository under GenBank accession numbers MH684491‐MH684522. The aligned GBS reads in the format of bam files were deposited in the NCBI repository under project ID PRJNA748759 and PRJNA748896.
