# Supplementary figures and images for: Demographic history and genetic diversity of wild African harlequin quail (Coturnix delegorguei delegorguei) populations of Kenya
Source: Ecol Evol. 2021 Dec 13;11(24):18562–74. doi: 10.1002/ece3.8458 (PMC8717324; doi:10.1002/ece3.8458)

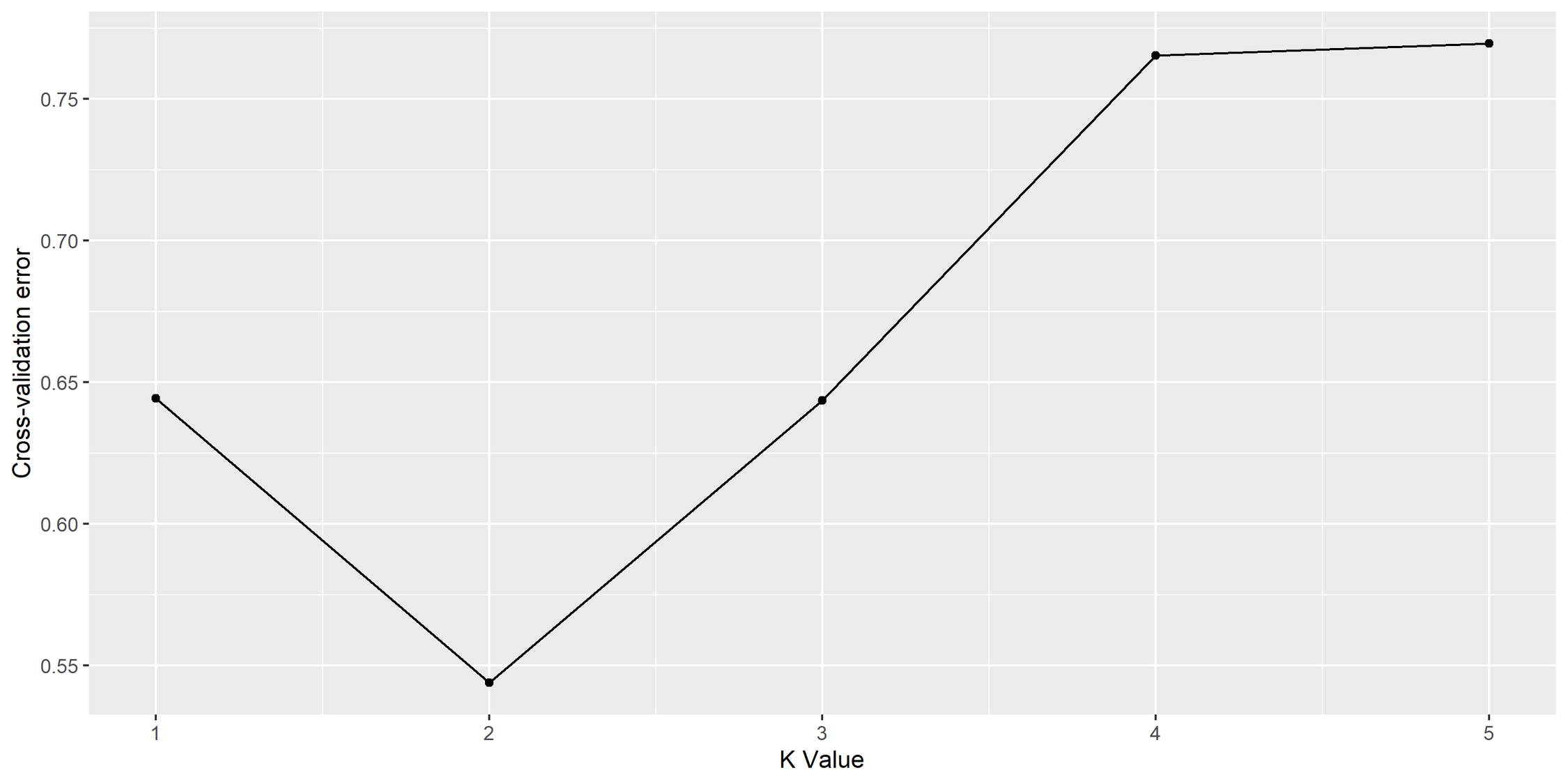

Supplement: Supplementary file 1 — Figure S1 [file ECE3-11-18562-s003.tif]

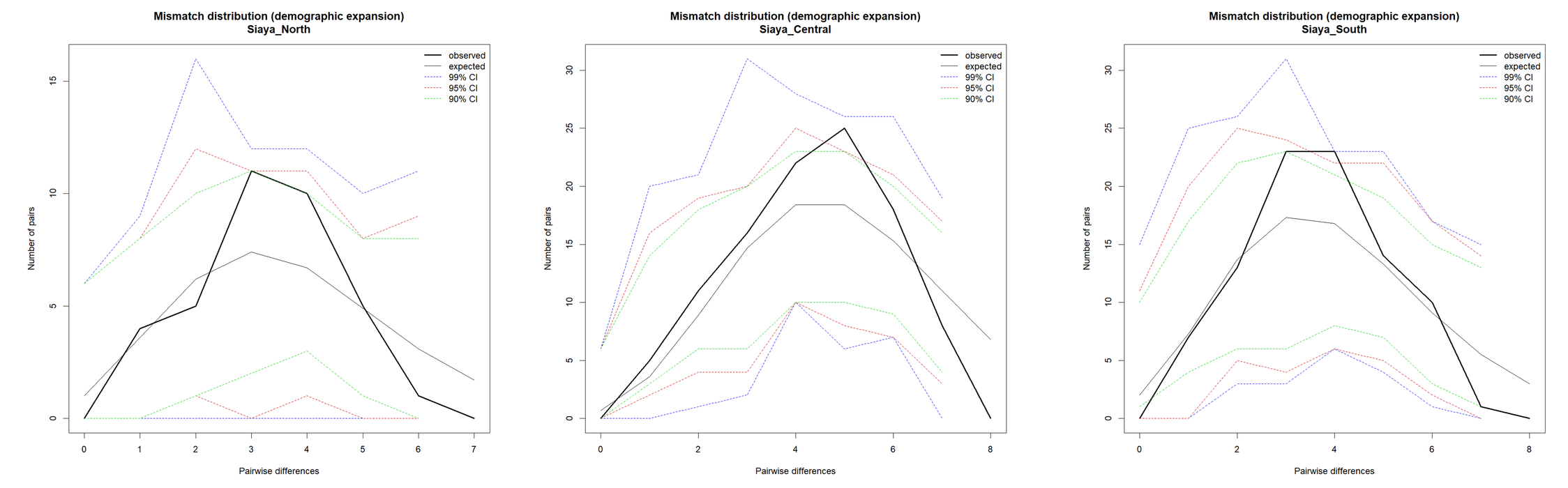

Supplement: Supplementary file 2 — Figure S2 [file ECE3-11-18562-s002.tif]
