# Supplementary material for: Demographic history and genetic diversity of wild African harlequin quail (Coturnix delegorguei delegorguei) populations of Kenya
Source: Ecol Evol. 2021 Dec 13;11(24):18562–74. doi: 10.1002/ece3.8458 (PMC8717324; doi:10.1002/ece3.8458)
Supplement: Supplementary file 3 — Tables S1 and S2 [file ECE3-11-18562-s001.docx]

**Supporting Information**

| Supporting Information Table S1: Phylogenetic tree reference quail mtDNA haplotypes | |
| --- | --- |
| **Reference Haplotype** | **Accession Number** |
| *Coturnix chinensis mitochondrial DNA (C. chinensis)* | AB073301.1 |
| *Coturnix coturnix haplotype 35 D-loop (C. coturnix haplot 35)* | KJ623801.1 |
| *Coturnix japonica* mitochondrion (*C. japonica* liu) | KX712089.1 |
| *Coturnix japonica* haplotype F1W1 (*C. japonica* haplo F1W1) | KF410830.1 |

| Supporting Information Table S2: Median network reference quail mtDNA haplotypes | |
| --- | --- |
| **Reference Haplotype** | **Accession Number** |
| *Coturnix chinensis* mitochondrial DNA | AB073301.1 |
| *Coturnix japonica* haplotype F2 control region | KF410831.1 |
| *Coturnix japonica* haplotype F3 control region | [KF410832.1](https://www.ncbi.nlm.nih.gov/nuccore/KF410832.1) |
| *Coturnix japonica* haplotype F4 control region | [KF410833.1](https://www.ncbi.nlm.nih.gov/nuccore/KF410833.1) |
| *Coturnix coturnix* haplotype W2 control region | KF410838.1 |
| *Coturnix japonica* haplotype W6 control region | KF410842.1 |
| *Coturnix coturnix* haplotype W9 control region | KF410845.1 |
| *Coturnix japonica* haplotype F1W1 | KF410830.1 |
